# Behavioral and Lifestyle Determinants of Poor Glycemic Control Among Adults with Type 2 Diabetes in Lesotho: Implications for Public Health in Low-Resource Settings

**DOI:** 10.3390/ijerph23010044

**Published:** 2025-12-29

**Authors:** Matseko Violet Tom Moseneke, Olufunmilayo Olukemi Akapo, Mirabel Kah-Keh Nanjoh, Sibusiso Cyprian Nomatshila

**Affiliations:** 1 Division of Public Health, Faculty of Medicine and Health Sciences, Walter Sisulu University, Mthatha 5100, South Africa; 222230452@mywsu.ac.za (M.V.T.M.); snomatshila@wsu.ac.za (S.C.N.); 2 Department of Laboratory Medicine and Pathology, Faculty of Medicine and Health Sciences, Walter Sisulu University, Mthatha 5100, South Africa; oakapo@wsu.ac.za

**Keywords:** type 2 diabetes mellitus, glycemic control, behavioral determinants, lifestyle modification, Lesotho

## Abstract

Type 2 diabetes mellitus (T2DM) is a growing public health challenge worldwide, disproportionately affecting populations in low- and middle-income countries (LMICs). Poor glycemic control contributes significantly to the global burden of non-communicable diseases (NCDs), increasing morbidity, mortality, and healthcare costs. Understanding behavioral and lifestyle determinants is critical for designing effective public health strategies, particularly in resource-limited settings such as Lesotho. A cross-sectional population-based study was conducted among 184 adults with T2DM attending the out-patient department of Maluti Adventist Hospital, Lesotho. Data was collected using a structured questionnaire and analyzed descriptively with SPSS 26 Variables assessed included sociodemographic, dietary practices, physical activity, behavioral risk factors and self-care knowledge. Participants were predominantly aged 45–69 years (65.2%), with an equal sex distribution. Hypertension was the most prevalent comorbidity (65.2%). Risk factor exposure was widespread, 100% consumed fewer than five daily servings of fruits/vegetables, 95.1% reported insufficient physical activity, and 88.0% had elevated blood pressure. Overall, 86.4% had three or more NCD risk factors. Knowledge levels were intermediate, with 33.2% scoring poor, 52.7% moderate, and only 14.1% good. Glycemic control was suboptimal, with 40.8% uncontrolled. This study highlights the urgent public health need to address lifestyle and behavioral determinants of poor glycemic control in Lesotho. Tailored interventions focusing on dietary education, physical activity promotion, and routine monitoring are essential to reduce NCD risks and improve outcomes. The findings have broader implications for achieving Sustainable Development Goal 3.4 on reducing premature NCD mortality in LMICs. Strengthening culturally sensitive health promotion, community-based interventions, and integrated chronic disease care models could significantly advance diabetes prevention and control in low-resource settings.

## 1. Introduction

Diabetes mellitus (DM) is a heterogeneous metabolic disorder characterized by chronic hyperglycemia due to impaired insulin secretion, insulin resistance, or both [1,2,3]. The two most common forms are type 1 diabetes mellitus (T1DM), an autoimmune condition leading to β-cell destruction and absolute insulin deficiency, and type 2 diabetes mellitus (T2DM), which results from impaired β-cell function combined with peripheral insulin resistance [4,5]. While T1DM typically presents in childhood, T2DM accounts for the majority of global cases and is closely linked to obesity, sedentary lifestyles, and poor dietary patterns. The burden of diabetes has risen dramatically worldwide. In 2014, the World Health Organization estimated that 422 million adults were living with diabetes, with global prevalence increasing from 4.7% in 1980 to 8.5% in 2014, disproportionately affecting low- and middle-income countries [6,7,8]. The International Diabetes Federation projects that the number of cases will reach 629 million by 2045 if current trends continue [9,10,11]. This rising prevalence is compounded by severe microvascular and macrovascular complications, including retinopathy, nephropathy, neuropathy, cardiovascular disease, and stroke [12,13,14].

In sub-Saharan Africa, the rapid pace of urbanization and adoption of Western lifestyles has accelerated the diabetes epidemic [15,16]. Lesotho reflects this trend, with the District Health Information System (DHIS) reporting consistently high numbers of new diabetes cases in recent years 11,671 in 2019, 9709 in 2020, 10,734 in 2021, and 9552 in 2022 [17,18,19]. Although patients at Maluti Adventist Hospital often receive regular treatment supplies, many are admitted with uncontrolled hyperglycemia, suggesting poor adherence, inadequate self-management, and behavioral challenges. This growing burden highlights the urgent need to address modifiable lifestyle factors such as diet, weight management, and physical activity, which remain central to both prevention and control of T2DM [20,21,22,23,24].

Given Lesotho’s struggle to meet Sustainable Development Goal 3 targets on reducing premature NCD mortality, strengthening knowledge, attitudes, and practices around lifestyle modification is critical. Understanding these factors among diabetic patients will inform interventions, policies, and education strategies to improve glycemic control, reduce complications, and mitigate the national disease burden.

## 2. Materials and Methods

This study employed a descriptive cross-sectional design and was conducted at the out-patient department of Maluti Adventist Hospital (MAH), located in Mapoteng village at the foothills of the Maluti Mountains in the Berea District of Lesotho. MAH, owned by the Seventh-Day Adventist Church, provides healthcare services to approximately 100,000 people across 264 villages, while the wider Berea District covers about 2222 km^2^ with an estimated population of 300,000.

### 2.1. Study Population and Sampling

**Figure 1:**
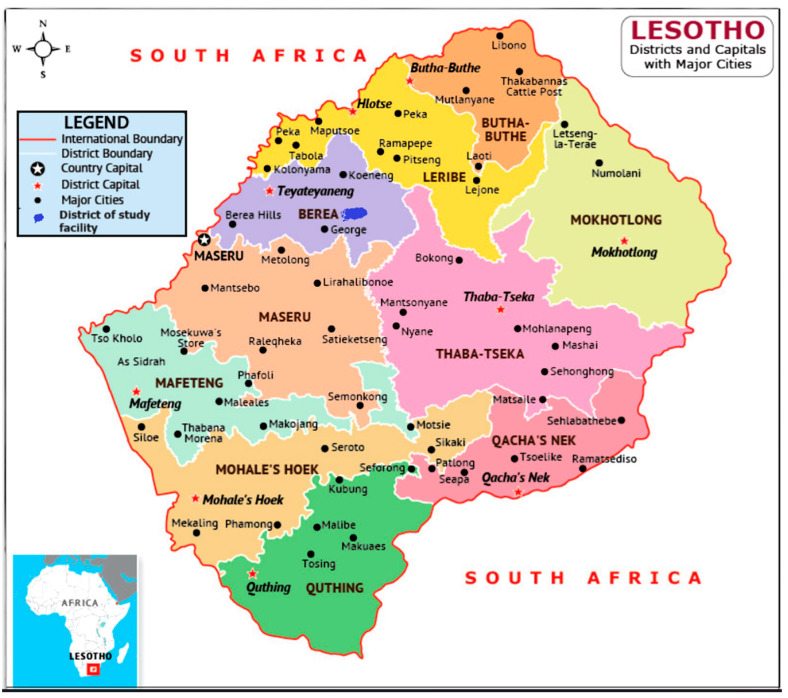
The Kingdom of Lesotho; Berea district.

The study included male and female adults aged 18 years and above with type 2 diabetes who attended the out-patient department of Maluti Adventist Hospital (MAH) in Berea, Lesotho (Figure 1) and provided informed consent. In addition to obtaining written informed consent, participants were counseled by diabetic nurses and nutritionists regarding the risks of recurrent hyperglycemia, including cardiovascular, renal, and ocular complications. Standard health-education sessions at Maluti Adventist Hospital form part of every outpatient diabetes review visit, reinforcing the importance of medication adherence, dietary modification, physical activity, and self-monitoring. These measures ensured that all participants were well informed of the clinical implications of poor glycemic control before and during study participation. Individuals with other medical conditions, visitors, and those not meeting the eligibility criteria were excluded.

### 2.2. Sample Size and Calculation

A total of 196 participants was required but only 184 agreed to participate. The expected proportion: P = 50%, Confidence level: 95% (Z_∝_ = 1.96) and the maximum error admitted by the researcher: e = 7% and the following formular was used:

$$n=\frac{Z_{\propto }^{2}*P(100-P)}{e^{2}}$$

#### 2.2.1. Inclusion Criteria

A consecutive sampling method was employed to include all eligible and consenting adults with type 2 diabetes who had been on treatment for at least 12 months and were attending follow-up visits at the Maluti Adventist Hospital outpatient department during the study period.

#### 2.2.2. Exclusion Criteria

All patients under the age of 18, those with T1DM and with gestational DM and patients who do not meet the described characteristics and the visiting patients who did not normally receive care at the MAH.

### 2.3. Data Collection Instrument and Method

Data was collected through face-to-face interviews using a structured questionnaire adapted from the WHO STEPwise approach to Non-Communicable Disease Risk Factor Surveillance (STEPS) instrument (WHO, 2008) and the validated Lewis (1994) Diabetes Self-Care and Perception Questionnaire [25,26]. The WHO STEPS tool was utilized to assess sociodemographic characteristics, physical activity, dietary practices, and behavioral risk factors, while the Lewis instrument captured diabetes-related knowledge, self-care behaviors, and treatment experiences. These instruments have been widely applied in epidemiological and behavioral studies of diabetes management, ensuring both content validity and cross-context comparability. Data was collected by the researcher at the out-patient department of Maluti Adventist hospital in Lesotho and around thirty minutes depending on the ability of the respondents to understand the questions.

All participants were under ongoing pharmacological management for type 2 diabetes as part of their regular outpatient follow-up at Maluti Adventist Hospital. The majority were prescribed oral hypoglycemic agents, primarily metformin (500–850 mg once or twice daily), consistent with Lesotho’s national diabetes treatment protocol and WHO essential medicines recommendations. A smaller subset, particularly those with long-standing or poorly controlled diabetes, received combination therapy involving metformin and insulin or other adjunct oral agents as directed by attending physicians. These treatment details were confirmed through medical record review during data collection. The study did not involve direct manipulation of therapy but observed patients under standard clinical management to explore behavioral and lifestyle correlates of glycemic control.

This study did not include direct measurement of clinical parameters such as systolic or diastolic blood pressure. Instead, comorbid hypertension was identified from participants’ routine outpatient records in accordance with hospital documentation practices and WHO STEPwise criteria.

#### 2.3.1. Validity

The instrument was translated into the language mostly spoken on the study site. The researcher translated the questionnaire into Sesotho to ensure consistency in its use when a Sesotho speaking participant was to be interviewed. The tool used for data collection was a validated instrument.

#### 2.3.2. Reliability

The data collection was done by the researcher and to further ensure reliability, the research instruments were administered by the researcher.

### 2.4. Data Management and Statistical Analysis

Microsoft Excel was used to handle and gather the data collected. The Statistical Package for Social Sciences (SPSS) software version 26 (SPSS Inc., Chicago, IL, USA) were used to analyze data. Categorical data was presented in the form of tables and figures and was presented as frequency (n) and proportions (percent). With a 95 percent confidence interval reporting. The information gathered was divided into three categories: demographics, knowledge, and attitudes and practices. Non-parametric statistics (median and interquartile range (IQR) were used to report numerical data that is not regularly distributed. The mean and standard deviation were used when reporting on regularly distributed data.

Glycemic control status was determined using each participant’s most recent HbA1c result documented within three months of the study, where HbA1c < 7.0% was classified as controlled and HbA1c ≥ 7.0% as uncontrolled, in accordance with WHO and ADA guidelines.

In line with hospital protocol, patients’ glycemic control was routinely monitored using glycated hemoglobin (HbA1c) values documented within three months of data collection. According to ADA (2024) and WHO (2023) criteria, HbA1c < 7.0% (53 mmol/mol) indicated controlled diabetes, whereas HbA1c ≥ 7.0% (53 mmol/mol) represented uncontrolled diabetes. Fasting and post-prandial blood glucose levels were also reviewed during clinical follow-ups, and any participant found to have persistently elevated glucose values was referred to the medical team for regimen adjustment and reinforcement of adherence counseling.

## 3. Results

A total of 184 adults with type 2 diabetes mellitus (T2DM) participated in this study, representing a 93.9% response rate from the initially targeted sample of 196. The sample comprised 93 females (50.5%) and 91 males (49.5%), demonstrating a nearly equal sex distribution (Table 1). The majority of participants were aged 45–69 years (65.2%), with a mean age of 64.0 ± 12.3 years, indicating that middle-aged and older adults formed the dominant study population. 57.6% resided in peri-urban areas, 31.5% in urban, and 10.9% in rural locations. Nearly all respondents (98.9%) lived in brick houses, while only two reported residing in non-permanent structures. Household energy use patterns revealed that gas (62.5%) and electricity (53.8%) were the predominant cooking sources, while 22.8% relied on wood and 2.7% on paraffin. Notably, more than one-third of participants reported using two or more energy sources, reflecting diverse socioeconomic circumstances across the study population.

**Figure 2:**
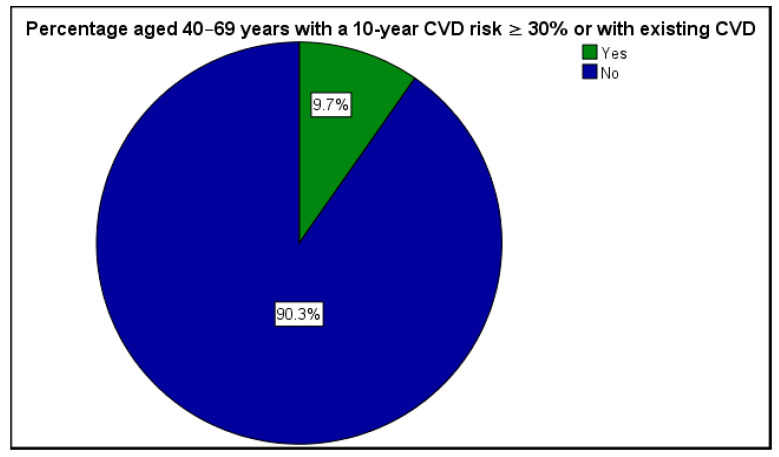
Ten–year CVD risk ≥10%.

As shown in Table 2, hypertension was the most prevalent comorbidity, affecting 65.2% of the study participants. Other common conditions included heart disease or stroke (13.0%) and hypercholesterolemia (4.3%), confirming the frequent coexistence of cardiovascular risks among individuals with T2DM. The 10-year cardiovascular disease (CVD) risk assessment indicated that a considerable proportion of participants fell within a moderate-to-high risk category (≥10%), particularly those aged above 60 years and with multiple metabolic risk factors (Figure 2).

The study identified widespread exposure to modifiable non-communicable disease (NCD) risk factors (Table 3). All participants (100%) consumed fewer than five servings of fruits and vegetables daily, while 95.1% reported insufficient physical activity, defined as less than 150 min of moderate-intensity activity per week. Tobacco use was relatively low (9.2%), the clustering of multiple risk factors was notable 86.4% of participants presented with three or more concurrent risk factors.

**Figure 3:**
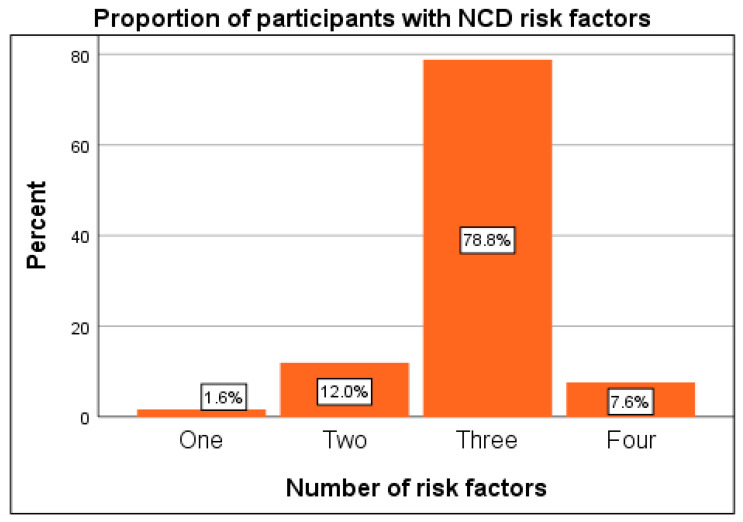
Participants with NCD risk factors.

Further analysis revealed that most (86.4%) participants had three or more risk factors. 7.6% of the participants had four risk factors while majority (78.8%) had three risk factors, followed by 22 (12%) with two risk factors. Only three (3; 1.6%) participants had one risk factor underscoring the complexity of lifestyle and metabolic challenges among adults with T2DM in this setting (Figure 3).

Table 4 summarizes self-care practices and perceptions related to diabetes management. Of the eight items assessed, six demonstrated good practice, one reflected poor adherence, and two indicated intermediate responses. The strongest agreement was observed for the statements emphasizing the importance of routine clinic attendance and dietary adherence, with most participants recognizing their role in achieving optimal diabetes control. Participants with HbA1c < 7.0% (53 mmol/mol) were categorized as having controlled diabetes, while those with HbA1c ≥ 7.0% (53 mmol/mol) were classified as having uncontrolled diabetes. This cutoff aligns with the American Diabetes Association (ADA, 2024) and World Health Organization (WHO, 2023) recommendations for optimal glycemic control among adults with type 2 diabetes.

**Figure 4:**
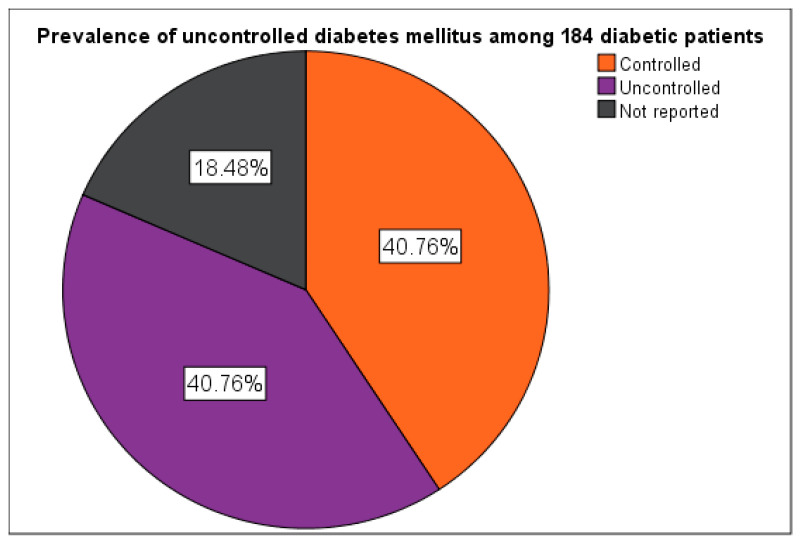
Prevalence of uncontrolled DM.

Among the 184 participants, 75 (40.76% 95% CI: 33.85%–47.96%) achieved glycemic control (HbA1c < 7.0%), 75 (40.76% 95%CI: 33.85%–47.96%) presented with uncontrolled diabetes (HbA1c ≥ 7.0%), and 34 (18.48% 95% CI: 13.39%–24.55%) had no recent HbA1c data available (Figure 4). Participants with HbA1c < 7.0% (53 mmol/mol) were categorized as having controlled diabetes, while those with HbA1c ≥ 7.0% (53 mmol/mol) were classified as having uncontrolled diabetes. This cutoff aligns with the American Diabetes Association (ADA, 2024) recommendations for optimal glycemic control among adults with type 2 diabetes.

## 4. Discussion

This study’s findings offer nuanced insights into the patterns of modifiable lifestyle practices among individuals with diabetes or at risk. Notably, among the eight assessed behaviors, six reflected strong practice, one showed poor engagement, and two demonstrated intermediate adherence. The singular poor practice participants’ disagreement with the value of regular urine checks for diabetes control highlights a critical gap in perceived self-care benefit. That finding aligns with broader evidence suggesting that patient perceptions significantly shape their engagement in self-monitoring behaviors [25,27,28,29]. The robust adoption of dietary adherence, medication compliance, and self-managed care behaviors demonstrates high perceived benefit and effective internal motivation. This phenomenon is well-supported by recent studies emphasizing the central role of self-efficacy and illness perception in fostering sustained diabetes self-care [30,31,32]. These cognitive constructs appear to empower individuals to proactively manage complex regimens, reinforcing our findings.

Meanwhile, neutral responses regarding lifestyle restrictions and regular clinic visits suggest ambivalence or uncertainty about these practices. These intermediate levels of adherence may be influenced by broader systemic or psychosocial dynamics. For instance, self-care behaviors are often moderated by socio-economic constraints, health literacy, and accessibility issues, especially in resource-limited settings [33]. Additionally, underlying diabetes-related distress can impede adherence, even when self-efficacy is high [34,35,36,37,38]. Overall, these findings underscore the critical importance of enhancing patient education around lesser-valued practices such as urine testing to improve perceived benefit. Interventions that strengthen self-efficacy and positively shape illness perception may help shift neutral to positive engagement in intermediate domains. Existing evidence supports that lifestyle interventions targeting diet, physical activity, and self-monitoring can significantly improve glucose control, quality of life, and cardiovascular outcomes [39,40,41,42]. Healthcare professionals should reframe and reinforce the value of regular urine or other monitoring tools, perhaps by contextualizing them within daily diabetes self-care routines. Interventions leveraging self-efficacy and positive illness framing could further solidify adherence across a broader range of self-care behaviors [27]. Enhanced accessibility to clinics, intuitive reminders, and culturally sensitive education could help alleviate neutrality toward clinic visits and perceptions of lifestyle limitations. Incorporating structured programs that integrate diet, exercise, and behavioral support has demonstrated meaningful improvements in glycemic control and wellbeing [43,44,45,46].

### Strengths and Limitations

This study’s strength lies in its detailed profiling of lifestyle adherence across multiple domains, illuminating both strengths and areas of neglect in self-care behavior. However, the cross-sectional design precludes causal inference and may underestimate dynamic changes over time. A limitation of this study is the absence of detailed clinical measurements, including systolic blood pressure and diastolic blood pressure values, body mass index and lipid levels, which would have strengthened the physiological context of glycemic control. Future studies should incorporate comprehensive clinical profiling to complement behavioral and lifestyle data, also longitudinal and interventional studies are warranted to evaluate how modifying self-efficacy, illness perception, and educational strategies can shift intermediate practices toward sustained engagement

## 5. Conclusions

Our findings illustrate a generally favorable self-care profile among participants, tempered by significant gaps in specific monitoring behaviors and ambivalent views on routine clinic attendance and lifestyle constraints. To optimize diabetes management, tailored interventions that reinforce perceived benefits, foster confidence in self-care, and address contextual health system barriers are essential. Embedding these strategies within a comprehensive, behaviorally informed framework holds promise for improving outcomes in diabetes populations.

### Future Research

This study provides a foundational understanding of the behavioral determinants influencing glycemic control among adults with type 2 diabetes in Lesotho. However, several areas warrant further investigation to deepen and broaden these insights.

First, longitudinal studies are needed to assess changes in lifestyle practices over time and to determine causal relationships between behavioral factors and glycemic outcomes. Understanding how behaviors evolve with age, treatment duration, and intervention exposure would inform more adaptive care models.

Second, qualitative research exploring patient beliefs, barriers, and facilitators of diabetes self-management could uncover nuanced psychosocial and cultural dynamics not captured in quantitative designs. In particular, exploring perceptions around urine glucose monitoring, clinic visits without symptoms, and perceived lifestyle restrictions would yield valuable insights for targeted intervention design. Third, future studies should incorporate objective clinical measures, such as HbA1c levels, BMI, and lipid profiles, to more accurately correlate self-reported practices with biological outcomes. Combining these with structured assessments of diabetes-related distress, health literacy, and self-efficacy could enhance predictive modeling of adherence behaviors. Additionally, interventional research evaluating the efficacy of behaviorally informed educational programs particularly those that emphasize the perceived benefits of under-practiced behaviors could help address existing gaps in diabetes self-management. Mobile health technologies, peer support structures, and culturally tailored messaging should be explored as scalable approaches to reinforce adherence in low-resource settings. Finally, expanding this research to diverse geographic regions and including larger, more heterogeneous samples would improve generalizability and help develop context-specific policies and programs aimed at reducing the burden of type 2 diabetes in sub-Saharan Africa and similar contexts.

## Figures and Tables

**Table 1:** Sociodemographic Characteristics of diabetic patients.

Characteristics	n (%), N = 184
Sex	
Male	91 (49.5)
Female	93 (50.5)
Total	184 (100)
Age groups (years)	
<45 years	5 (2.7)
45–69 years	120 (65.2)
>69 years	59 (32.1)
Age (years)/Mean (SD)	64 (12.3)
Residential location	
Rural	20 (10.9)
Peri-urban	106 (57.6)
Urban	58 (31.5)
Type of house	
Corrugated iron shack	1 (0.5)
Mudhouse	1 (0.5)
Brick house	182 (98.9)
Type of energy used for cooking	
Wood	42 (22.8)
Paraffin	5 (2.7)
Gas	115 (62.5)
Electricity	99 (53.8)

**Table 2:** Comorbidity among diabetic patients.

Comorbidity	n (%) N = 184
Hypertension	120 (65.2)
Hypercholesterolemia	8 (4.3)
Heart disease or a stroke	24 (13.0)

**Table 3:** Risk factors identified among patients.

Risk Factors of Interest	n (%), N = 184
Smoke and smokeless tobacco use	17 (9.2)
Eat less than five servings of fruit and vegetables on average per day	184 (100.0)
Insufficient physical activity (defined as <150 min of moderate-intensity activity per week	175 (95.1)
Raised BP (SBP ≥ 140 and or DBP ≥ 90 mmHg or currently on medication for raised BP)	162 (88.0)
Have three or more risk factors	159 (86.4)

**Table 4:** Practices of modifiable lifestyle factors.

Practice Items	Mean Score, N = 184
Controlling diabetes well imposes restrictions on all lifestyle	4
It is important to visit the diabetic clinic regularly, even in the absence of symptoms	4
Dietary adherence makes eating out difficult	3
It is difficult to remember to take all tablets at the times recommended by the doctor	3
It is just not possible to control diabetes properly and live in an acceptable way	3
Testing urine is an unpleasant task to have to undertake	3
It is hard to cut down on sugary and fatty foods	3
Regular urine checks enable the control of diabetes well	3

## Data Availability

The data presented in this study are not available due to ethical reason.

## Abbreviations

The following abbreviations are used in this manuscript:

DM	Diabetes Mellitus
SPSS	Statistical Package for Social Sciences
OPD	Outpatient Department
NCD	Non-Communicable Diseases
MAH	Maluti Adventist Hospital
DHIS	District Information health system
IDF	International diabetes federation
T1DM	Type one diabetes mellitus
T2DM	Type two diabetes mellitus
WHO	World health organization
